# A meta-analysis of the effects of strength training on physical fitness in dancers

**DOI:** 10.3389/fphys.2025.1511833

**Published:** 2025-02-27

**Authors:** Yunqing Yang, Nuannuan Deng, Xinggang Yang

**Affiliations:** ^1^ Department of Physical Education, Chongqing Vocational and Technical University of Mechatronics, Chongqing, China; ^2^ Department of Physical Education, Sichuan Fine Arts Institute, Chongqing, China; ^3^ College of Physical Education, Chongqing University, Chongqing, China

**Keywords:** ballroom, ballet, body composition, muscle strength, muscle power

## Abstract

**Background:**

Physical fitness is fundamental for successfully carrying out daily tasks and activities associated with dance. This meta-analysis aimed to evaluate the impact of strength training on various aspects of physical fitness in dancers.

**Methods:**

A comprehensive search of Web of Science Core Collection, PubMed, SPORTDiscus, SCOPUS, Cochrane library, CINAHL, and Embase was conducted until 10 December 2024, supplemented by hand-searches via Google Scholar and reference lists of included studies. Controlled trials that assessed the effects of strength training on at least one physical fitness measure in dancers were selected. Effect sizes (ES, Hedges’ g) were calculated using a random-effects model to compare experimental and control groups. Study quality was assessed using the Cochrane risk of bias tools.

**Results:**

A total of 15 studies, involving 351 dancers, met the eligibility criteria. The analyses revealed significant moderate to large effects of strength training on muscle strength (ES = 1.84; 95% CI: 0.90 to 2.77; p < 0.001) and muscle power (ES = 0.64; 95% CI: 0.30 to 0.98; p < 0.001). Non-significant effects (all p > 0.05) were found for body mass (ES = 0.13; 95% CI: −0.32 to 0.58; p = 0.572), body fat percentage (ES = 0.08; 95% CI: −0.04 to 0.61; p = 0.754), cardiorespiratory endurance (ES = 0.28; 95% CI: −0.48 to 1.04; p = 0.469), and flexibility (ES = 0.37; 95% CI: −0.06 to 0.79; p = 0.090).

**Conclusion:**

The findings indicate that strength modalities, including resistance training, plyometric training, weight training, and combined programs, positively impact muscle strength and power in dancers. However, future research should explore the effective training parameters (e.g., frequency, session length, intensity, and specificity of strength exercises) necessary to improve not only strength and power but also other components of physical fitness in dancers.

**Systematic Review Registration:**

https://www.crd.york.ac.uk/prospero/display_record.php?RecordID=596550, Identifier CRD42024596550.

## Introduction

Dance occupies a unique space between sport and artistic expression, combining aesthetics with physical performance. As artistic athletes, dancers need exceptional fitness to execute complex moves and routines (Guarino, 2015). However, research indicates that dancers often have lower muscle strength, endurance, and jump performance compared to other athletes (Allen and Wyon, 2008; Angioi et al., 2009a). Similar to other sports, dance demands physical efficiency and excellence, but it also emphasizes the visual elegance essential to choreographed routines (Malkogeorgos et al., 2013). Moreover, it has been proposed that enhanced physical fitness is essential for excelling in the qualitative dimensions of dance performance such as skills, whole-body coordination, and precision in movement (Angioi et al., 2009a; Angioi et al., 2009b; Koutedakis et al., 2009). For example, maintaining muscle strength is considered vital for supporting technical execution and preventing injuries among dancers (Moita et al., 2017). Contemporary dance often involves dynamic movements such as kicks and leaps, which demand a combination of power and flexibility (Deighan, 2005; Skopal et al., 2020). Meanwhile, cardiorespiratory endurance plays a key role in enhancing both the aesthetic and technical aspects of dance, while also promoting overall health, increasing stamina, and meeting the intense physical demands of rehearsals and performances (Needham-Beck et al., 2019). Dance training alone is often deemed inadequate to meet the physical demands of the discipline, yet dancers rarely incorporate cross-training from other athletic fields (Rafferty, 2010; Skopal et al., 2020; Sanders et al., 2021). Therefore, adopting effective training strategies could bridge the gap between physical preparation and performance demands, address musculoskeletal imbalances, and enhance key aspects of performance.

Strength training is widely employed to improve physical fitness, performance, and overall athletic ability. A meta-analysis by Thiele et al. (2020) found that strength training significantly increased lower-limb maximal strength in rowers (standardized mean difference [SMD] = 0.42). Similarly, Balsalobre-Fernández et al. (2016) reported a substantial improvement in running economy among runners (SMD = −1.42) following strength training. Additionally, strength training demonstrated a moderate positive effect on time-trial performance in Olympic time-based sports (effect size [ES] = 0.59). Behringer et al. (2011) highlighted its efficacy in improving motor performance in children and adolescents, with ESs ranging from 0.52 to 0.99. A systematic review by Cid-Calfucura et al. (2023) further emphasized the benefits of strength training, showing improvements in strength, power, flexibility, and balance among Olympic combat sports athletes. Numerous individual studies also corroborate the role of strength training in enhancing various aspects of physical fitness in athletes (Naclerio et al., 2013; Rodríguez-Rosell et al., 2017; Hermassi et al., 2020). Notably, a recent meta-analysis evaluated the effects of strength and conditioning on physical qualities and aesthetic competence in dance populations, revealing significant improvements in upper and lower body strength (ES = 0.98–1.59), lower body power (ES = 0.90), and flexibility (ES = 0.86). However, this meta-analysis was limited to dancers aged over 16 years. Experimental studies have also investigated the effects of various strength training protocols on younger dancers. For example, De Leonardis and Greco (2020) demonstrated that an 8-week plyometric training program enhanced muscle power in young female dancers. Similarly, Ávila-Carvalho et al. (2022) observed improved jumping performance in young ballet dancers following 16 weeks of lower-limb strength training. With this in mind, further evidence on the effects of strength training on dancers’ physical fitness is warranted. A meta-analysis, which aggregates sample sizes from various studies, can provide high-quality evidence and offer new insights for practitioners, aiding in evidence-based decisions regarding the implementation of strength training (Morrell et al., 2016). Therefore, the objective of this study was to assess the effects of strength training on physical fitness parameters through a meta-analysis of the available literature.

## Methods

This meta-analysis was conducted in accordance with the PRISMA checklist for systematic reviews and meta-analysis (Page et al., 2021). Moreover, before conducting this study, the protocol was preregistered in PROSPERO to ensure methodological rigor and prevent duplication (Identifier: CRD42024596550; https://www.crd.york.ac.uk/prospero/display_record.php?RecordID=596550).

### Search strategy

A systematic search was conducted across seven electronic databases (Web of Science Core Collection, PubMed, SPORTDiscus, SCOPUS, Cochrane library, CINAHL, and Embase) to locate relevant studies from their inception until 10 December 2024. The selected databases were chosen for their extensive coverage of topics in physical activity, sports science, and related fields, as well as their access to high-quality sources. Previous reviews and meta-analyses (Slimani et al., 2018; Hughes et al., 2023; Ngo et al., 2024) were used to help define our search strategy. The Boolean search strategy utilized the operators “AND” and “OR” to combine and refine search terms: (danc* OR ballet OR salsa OR ballroom OR “hip-hop” OR jazz OR tap OR cha-cha OR rumba OR samba OR flamenco OR tango OR waltz OR folk) AND (“strength training” OR “resistance training” OR “weight training” OR “power training” OR “plyometric training” OR “complex training” OR “compound training” OR “neuromuscular training”). Following the formal systematic searches, additional hand-searches were conducted using Google Scholar and by reviewing the reference lists of included studies to identify relevant works. The search strings for each database are provided in Supplementary Appendix S1.

### Selection criteria

The present meta-analysis included studies that followed the PICOS framework (Liberati et al., 2009). The inclusion criteria were as follows: (a) participants were dancers of any age, sex, or dance genre; (b) studies involved strength training programs lasting more than 2 weeks; (c) included an active control group; (d) measured at least one physical fitness parameter (e.g., power, strength, flexibility) before and after the intervention; (e) were controlled trials published in peer-reviewed journals; and (f) were written in English to avoid translation challenges.

Studies were excluded if they met the following criteria: (a) lacked original data (e.g., protocols, reviews, or patents); (b) included dancers with specific injuries (e.g., knee ligament injuries); (c) did not use a control group to assess the impact of strength training; (d) failed to report outcomes related to physical fitness; or (e) integrated strength training programs with psychological methods (e.g., self-talk).

Two independent reviewers (YY, ND) assessed potentially relevant articles for eligibility by reviewing titles, abstracts, and full texts, based on the established inclusion and exclusion criteria (Table 1). If YY and ND disagreed on the inclusion or exclusion of an article, a third author (XY) was consulted for a final decision.

**TABLE 1:** Eligibility criteria according to the PICOS conditions.

Category	Inclusion criteria	Exclusion criteria
Population	Healthy male or female dancers	Participants from other sports
Intervention type	Strength training (e.g., resistance training, plyometric training)	Intervention not involving strength training
Comparator	Active control group	No control or passive control group
Outcome	At least one measure of physical fitness (e.g., strength, power, flexibility)	The data were not reported in means and standard deviations for the intervention and control groups at pre- and post-test
Study design	Controlled trials	Case study, observational study

### Data extraction

The first reviewer (YY) extracted data from the selected studies using a standardized form developed in Microsoft Excel, while the second reviewer (ND) independently cross-checked all the extracted information to ensure accuracy. In cases where discrepancies arose between the two reviewers, the study information was revisited for verification. The extracted data included the following: (1) general publication details (e.g., author names, publication year); (2) participant information (e.g., sample size, age, dance genre); (3) intervention specifics (e.g., duration, frequency, session length); and (4) outcome variables (e.g., power, strength, flexibility). For the meta-analysis, only original research papers that provided data compatible for calculations and used standardized outcome measures were selected.

### Risk of bias assessment

The risk of bias for each randomized controlled trial (RCT) included in the study was carefully assessed using the most recent version of the Cochrane risk of bias tool for randomized trials (RoB-2) (Flemyng et al., 2023). For non-RCTs, the evaluation was conducted using the Risk Of Bias In Non-randomized Studies of Interventions (ROBINS-I) tool (Sterne et al., 2016). Two independent reviewers (YY, ND) conducted the assessments. In instances where the reviewers disagreed, a consensus meeting was convened, and a third assessor (XY) provided an additional rating to resolve the differences and reach an agreement.

### Statistical analyses

All statistical analyses were conducted using Comprehensive Meta-Analysis software (Version 3.0; Biostat, Englewood, NJ, USA), which facilitated tasks such as the calculation of ESs, heterogeneity assessments, and the generation of forest plots. A meta-analysis was conducted when the outcome measure included sufficient data (at least three studies) (Deng et al., 2023; Deng et al., 2024). Many of the included studies had small sample sizes (fewer than 20 participants), necessitating an adjustment for small sample calculations (Anderson et al., 2017). Consequently, Hedges’ g ESs were calculated using pre- and post-intervention data to evaluate performance outcomes in both the experimental and control groups. In studies with multiple intervention groups, the control group’s sample size was proportionally divided to facilitate comparisons among all subjects (Higgins et al., 2008). The meta-analysis employed a random effects model for continuous data, incorporating inverse variance and a 95% confidence interval. The ES values obtained were classified according to the following scale: trivial (ES < 0.2), small (0.2 ≤ ES ≤ 0.6), moderate (0.6 < ES ≤ 1.2), large (1.2 < ES ≤ 2.0), very large (2.0 < ES ≤ 4.0), and extremely large (ES > 4.0) (Hopkins et al., 2009). To assess heterogeneity among studies, the I^2^ statistic was used, expressed as a percentage ranging from 0 to 100, indicating the extent of variability attributable to heterogeneity rather than random chance. Values below 25% signified low heterogeneity, those between 25% and 75% indicated moderate heterogeneity, and values exceeding 75% suggested high heterogeneity (Higgins and Thompson, 2002). The extended Egger’s test was conducted to assess the potential risk of publication bias among the studies (Egger et al., 1997), with a significance threshold set at p < 0.05 to establish statistical relevance.

## Results

### Study selection

**FIGURE 1:**
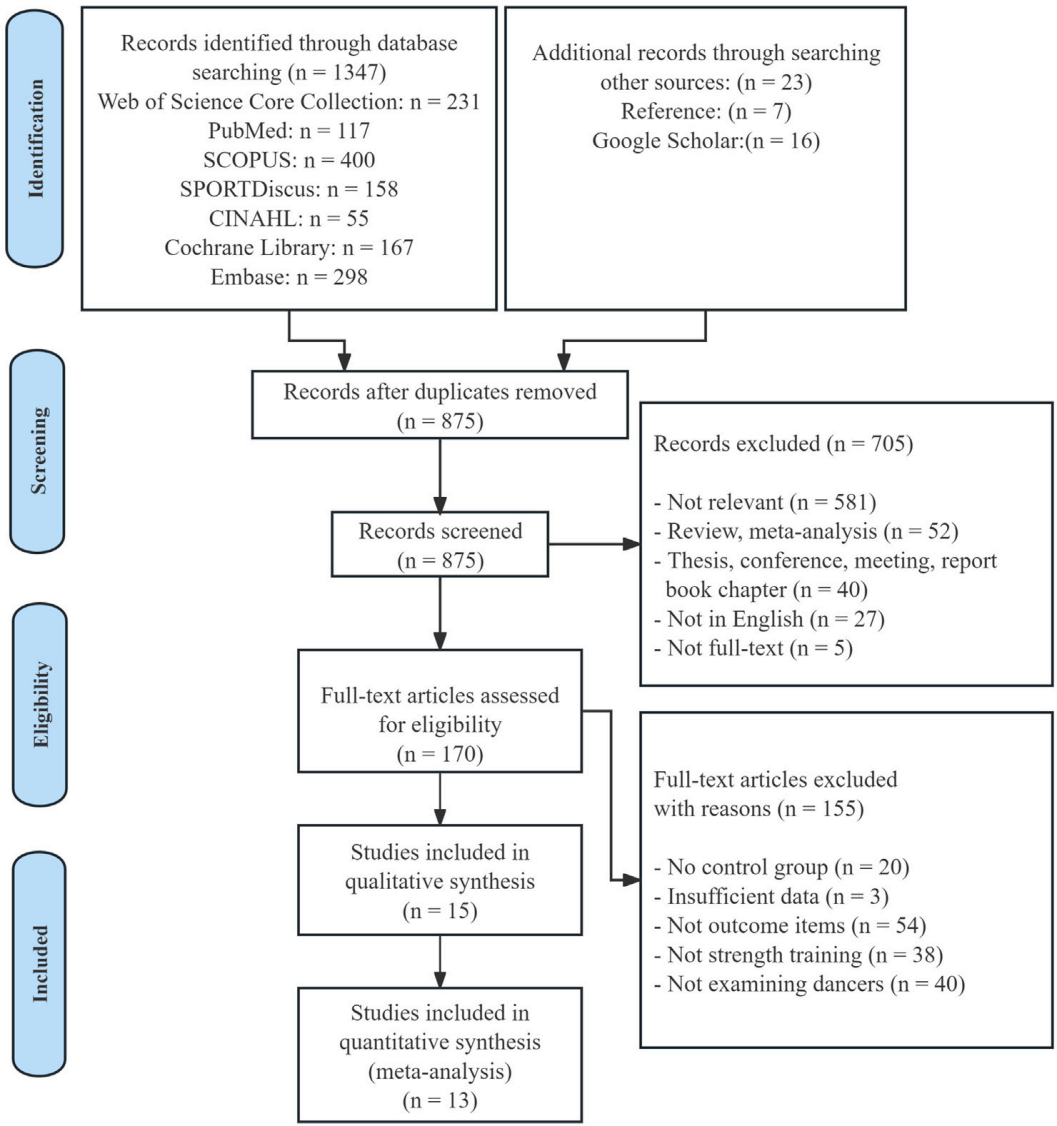
Flow diagram of the study selection.

As shown in Figure 1, the initial database search across multiple platforms identified 1,347 documents, supplemented by 23 studies found through manual searches of reference lists and Google Scholar. After removing duplicates, 875 unique records remained for initial screening. Titles and abstracts of these records were reviewed, narrowing the selection to 170 articles for full-text assessment. Each article was carefully evaluated against the inclusion criteria, leading to the exclusion of 155 studies due to issues such as inadequate study design, irrelevant outcomes, or insufficient data. Consequently, 15 studies met the inclusion criteria, with 13 included in the final meta-analysis.

### Methodological quality

**FIGURE 2:**
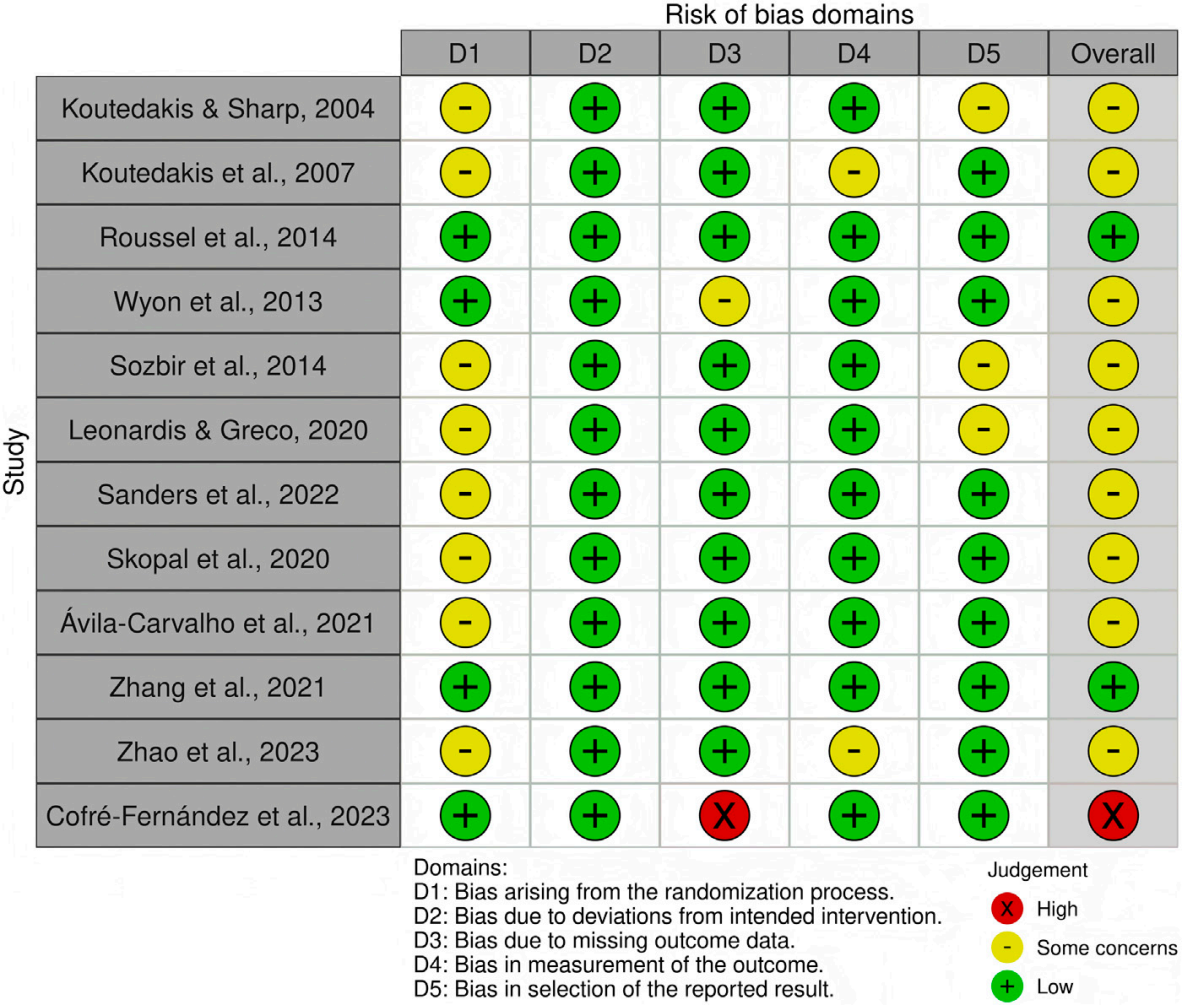
RoB-2 assessments. Created using Robvis tool.

**FIGURE 3:**
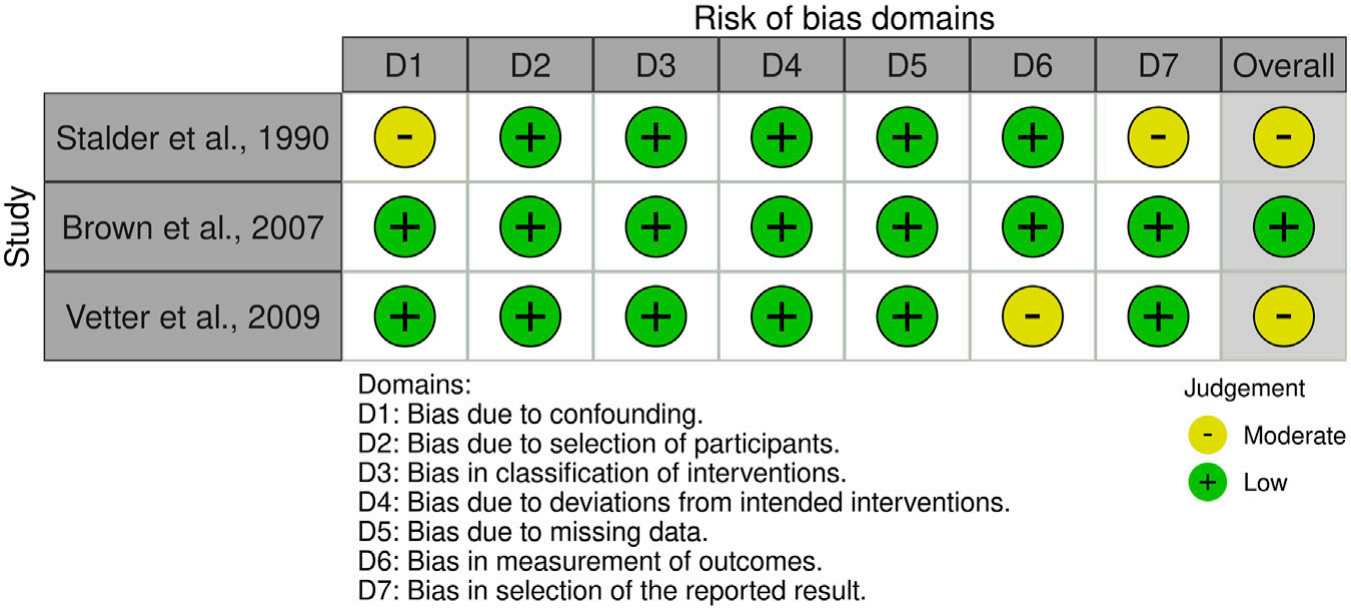
ROBINS-I assessments. Created using Robvis tool.

Twelve RCTs were assessed using the RoB-2 tool, while three non-RCTs were evaluated with the ROBINS-I framework. As depicted in Figures 2, 3, 13 studies presented a moderate risk of bias or some concerns, with only two categorized as having a low risk of bias. Figure 2 provides an overview of the RoB-2 assessment results. Of the RCTs, only three detailed their randomization sequence generation methods, whereas the remaining eleven did not provide sufficient descriptions of their randomization procedures. Three RCTs exhibited a high risk of bias and some concerns due to missing data, with dropout rates of approximately 10% and over 15%, respectively. Three RCTs raised concerns regarding the selection of reported outcomes, while two others had bias related to outcome measurements. Figure 3 visually represents the ROBINS-I findings. One non-RCT showed a moderate risk of bias due to selection bias, while two others had moderate risk stemming from outcome measurement and outcome reporting biases.

### Study characteristics

Table 2 provides a detailed overview of the primary characteristics of both the participants and the interventions involved in the studies. The studies included a total of 351 dancers from various styles, including ballet, contemporary, modern, jazz, ballroom, and hip-hop, with participants aged between 11 and 27 years. Among these studies, ten focused exclusively on females (Stalder et al., 1990; Koutedakis and Sharp, 2004; Brown et al., 2007; Vetter and Dorgo, 2009; Wyon et al., 2013; Sozbir et al., 2014; Skopal et al., 2020; De Leonardis and Greco, 2020; Sanders et al., 2020; Cofré-Fernández et al., 2023), while five assessed both genders (Koutedakis et al., 2007; Roussel et al., 2014; Zhang et al., 2021; Ávila-Carvalho et al., 2022; Zhao, 2023). The training modalities identified in these studies included lower limb strength training (Ávila-Carvalho et al., 2022; Zhao, 2023), resistance training (Koutedakis and Sharp, 2004; Sanders et al., 2020; Vetter and Dorgo, 2009), plyometric training (Brown et al., 2007; De Leonardis and Greco, 2020), weight training (Stalder et al., 1990; Brown et al., 2007), neuromuscular training (Zhang et al., 2021; Cofré-Fernández et al., 2023), and combined training (Koutedakis et al., 2007; Skopal et al., 2020; Roussel et al., 2014). The training interventions lasted between 6 and 16 weeks, with participants training two to three times per week. Each session ranged from 20 to 75 min. In terms of exercise volume, the interventions included between 2 and 10 sets per exercise, with each set consisting of 5–20 repetitions.

**TABLE 2:** Characteristics of the studies examined in the present meta-analysis.

Study	Participant characteristics				Intervention characteristics				Outcomes	Key findings
	Sample	Age	Level	Dance genre	Training method	Dur (weeks)	Freq (per week)	SL (min)		
Stalder et al. (1990)	14F	EG: 23.32 ± 4.3, CG: 20.4 ± 3.25	Collegiate	Ballet	EG: weight training (3 sets × 10 repetitions); CG: ballet technique class	9	3	NR	Muscle strength (leg extension), flexibility (anterior hip)	Leg extension→, anterior hip→
Koutedakis and sharp (2004)	22F	25 ± 1.3	Professional	Ballet	EG: quadriceps and hamstring strength training (5–6 sets of 3–4 exercises each, 8 repetitions in each exercise); CG: regular dancing	12	3	50	Body composition (body mass)	Body mass→
Koutedakis et al. (2007)	27F/5M	19 ± 2.2	Professional	Modern	EG: aerobic training + free-weight exercises) (5–6 sets of 3–4 exercises each, 8 reps in each exercise); CG: school curriculum	12	3	50	Muscle strength (leg strength), cardiorespiratory endurance (VO2max), flexibility (hamstring flexibility)	leg strength ↑, VO2max ↑, hamstring flexibility ↑
Brown et al. (2007)	12F	18-23	Collegiate	NR	EG1: plyometric training (3 sets × 8 repetitions); EG2: weight training; CG: regular dance regimens	6	2	30-45	Body composition (body mass, body fat%); muscle strength (leg press), power (VJ)	EG1 and, EG2: body mass →, body fat% →, leg press ↑, EG1: VJ ↑, EG2: VJ→
Vetter and Dorgo (2009)	18F	20.5 ± 2.0	Collegiate	Ballet, modern, and jazz	EG: resistance training (2 sets × 10 repetitions); CG: dance classes	8	3	60	Muscle strength (leg extension)	Leg extension↑
Wyon et al. (2013)	39F	17 ± 0.52	Moderately trained	Ballet	EG1: strength training (2 sets × 5-8 repetitions); E.G.,2: low-intensity stretching; E.G.,3: moderate-intensity stretching, EG4: high-intensity stretching; CG: passive stretches	6	NR	NR	Flexibility (ROM)	ROM↑
Roussel et al. (2014)	38F/6M	EG: 19.9 ± 2.0 CG: 19.6 ± 2.4	Pre-professional	Ballet	EG: aerobic endurance, strength, proprioception and motor control training; CG: health promotion intervention	16	2act	75	Body composition (body mass), muscle power (SBJ), cardiorespiratory endurance (VO2max)	Body mass →, SBJ →, VO2max →
Sozbir et al. (2014)	27F	20.14 ± 0.95	Collegiate	Contemporary	EG: plyometric training (2–5 sets × 6-15 reps); CG: routine contemporary dance	6	2	NR	Body composition (body mass), muscle power (VJ)	Body mass →, VJ ↑
De Leonardis and Greco (2020)	16F	EG: 15.0 ± 2.3 CG: 14.3 ± 1.3	NR	Contemporary	EG: plyometric training (3 sets × 8 reps); CG: regular dance regimen	8	2	30-45	Body composition (body fat %, body mass), muscle power (CMJ)	CMJ ↑, body fat % →, body mass →
Sanders et al. (2020)	16F	19.3 ± 1.3	Collegiate	NR	EG: resistance-training (3 sets × 10- 12 reps); CG: dance training	8	3	NR	Body composition (body mass, body fat%), muscle power (VJ), muscle strength (1RM squat), cardiorespiratory endurance (VO2max)	Body mass →, body fat% ↑, VJ ↑, 1 RM squat ↑, VO2max →
Skopal et al. (2020)	11F	19.0 ± 2.0	Intermediate	Contemporary	EG: rhythmic gymnastics- based supplementary training (plyometric training); CG: usual training	8	2	60	Muscle power (leg peak kicking torque), flexibility (ROM)	leg peak kicking torque ↑, ROM ↑
Zhang et al. (2021)	21F/21M	17-23	Elite	Ballroom	EG: neuromuscular training (3sets × 10 reps/30–60s); CG: dance training routines	10	3	60	Balance (Y balance test)	Y balance test ↑
Ávila-Carvalho et al. (2022)	19F/5M	EG: 12.43 ± 1.45; CG: 13.00 ± 1.49	NR	Ballet	EG: lower-limb strength training (3-6sets × 3-10 reps); CG: regular dance training	16	2	20	Body composition (body mass), muscle power (CMJ)	Body mass →, CMJ ↑
Zhao (2023)	10F/10M	19-22	Collegiate	Ballroom	EG: lower-limb strength training; CG: regular dance training	12	3	30	Muscle power (VJ)	VJ ↑
Cofré-Fernández et al. (2023)	14F	15-20	Competitive	Hip-hop and jazz	EG: neuromuscular training (2–3 sets × 10-20 reps/30–60s); CG: habitual dance training	6	3	30	Balance (Y balance test)	Y balance test ↑

EG, experimental group; CG, control group; NR, not reported; yrs, years; M, male; F, female; Dur, Duration; Freq, frequency; reps, repetitions; SL, session length; VJ, vertical jump; CMJ, countermovement jump; SBJ, standing broad jump; 1RM, 1-repetition max; ROM, range of motion, VO2max, maximal oxygen consumption; ↑, significant within-group improvement; ↔, non-significant within-group.

Notably, only two studies investigated the effects of neuromuscular training on balance in dancers, and thus, they were excluded from the meta-analysis. Zhang et al. (2021) implemented a 10-week neuromuscular training program with three sessions per week, which significantly improved balance performance in 42 elite collegiate ballroom dancers (aged 17–23 years). The neuromuscular training group scored significantly higher on the post-test (392 ± 36) compared to the control group (329 ± 23). Similarly, Cofré-Fernández et al. (2023) evaluated the effects of a 6-week neuromuscular training program, also comprising three sessions per week, on balance in 14 female competitive hip-hop and jazz dancers (aged 12–20 years). The experimental group exhibited significant improvements in Y-balance test scores (p = 0.01–0.02; ES = 0.58–0.63).

### Meta-analyses results

#### Body composition

**FIGURE 4:**
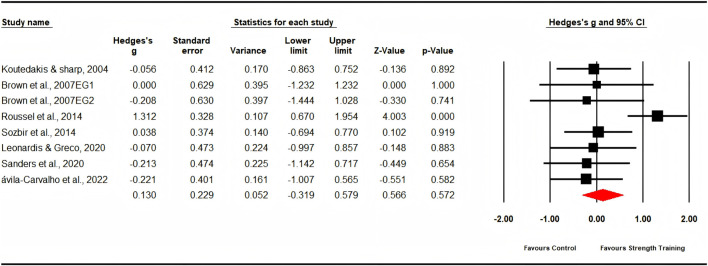
Forest plot of changes in body mass in dancers participating in strength training compared to controls. Values shown are effect sizes (Hedges’ g) with 95% confidence intervals (CI). The size of the plotted squares reflects the statistical weight of the study. EG = experimental group.

**FIGURE 5:**
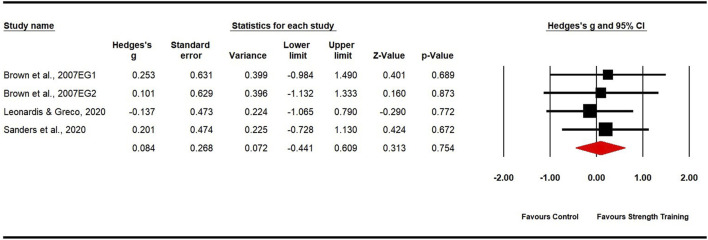
Forest plot of changes in body fat percentage in dancers participating in strength training compared to controls. Values shown are effect sizes (Hedges’ g) with 95% confidence intervals (CI). The size of the plotted squares reflects the statistical weight of the study. EG = experimental group.

Seven studies evaluated body mass, comprising six experimental groups and five control groups (pooled n = 167). The findings revealed a non-significant, trivial effect of strength training on body mass (ES = 0.13; 95% CI: −0.32 to 0.58; p = 0.572; Figure 4), with moderate heterogeneity observed (I^2^ = 52.65%). The Egger’s test yielded a p-value of 0.125. Additionally, three studies assessed body fat percentage, involving four experimental groups and three control groups (pooled n = 50). The results indicated a non-significant, trivial effect of strength training on body fat percentage (ES = 0.08; 95% CI: −0.04 to 0.61; p = 0.754; Figure 5), with low heterogeneity observed (I^2^ = 0.00%). The Egger’s test for this analysis produced a p-value of 0.555.

#### Muscle strength

**FIGURE 6:**
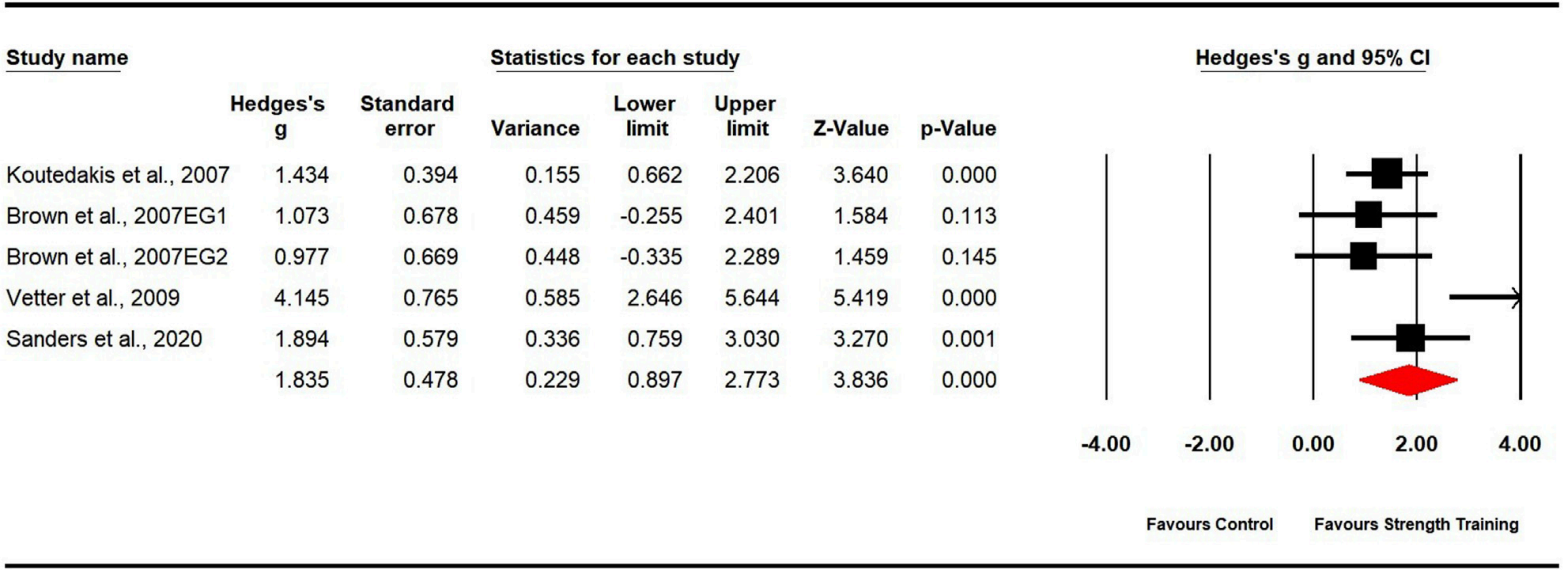
Forest plot of changes in muscle strength in dancers participating in strength training compared to controls. Values shown are effect sizes (Hedges’ g) with 95% confidence intervals (CI). The size of the plotted squares reflects the statistical weight of the study. EG = experimental group.

Four studies evaluated muscle strength, including five experimental groups and four control groups (pooled n = 87). The analysis demonstrated a significant, large effect of strength training on muscle strength (ES = 1.84; 95% CI: 0.90 to 2.77; p < 0.001; Figure 6). Moderate heterogeneity was observed (I^2^ = 68.77%), and the Egger’s test yielded a p-value of 0.490. To explore the sources of heterogeneity, we removed one outlier (Vetter and Dorgo, 2009), which adjusted the ES to 1.40 (95% CI: 0.88–1.93; p < 0.001) and reduced heterogeneity to 0%.

#### Muscle power

**FIGURE 7:**
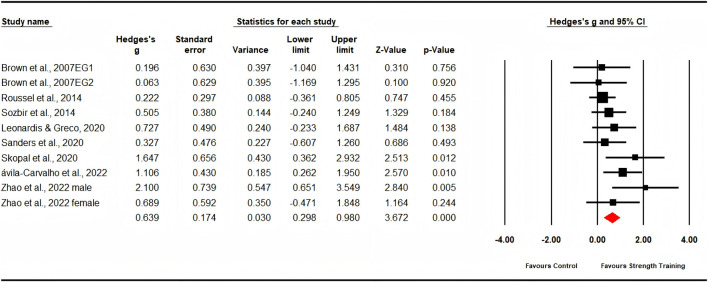
Forest plot of changes in muscle power in dancers participating in strength training compared to controls. Values shown are effect sizes (Hedges’ g) with 95% confidence intervals (CI). The size of the plotted squares reflects the statistical weight of the study. EG = experimental group.

Nine studies evaluated muscle power, comprising eleven experimental groups and nine control groups (pooled n = 176). The analysis showed a significant, moderate effect in favor of strength training (ES = 0.64; 95% CI: 0.30 to 0.98; p < 0.001; Figure 7). Low heterogeneity was observed (I^2^ = 20.27%), and the Egger’s test produced a p-value of 0.124.

#### Cardiorespiratory endurance

**FIGURE 8:**
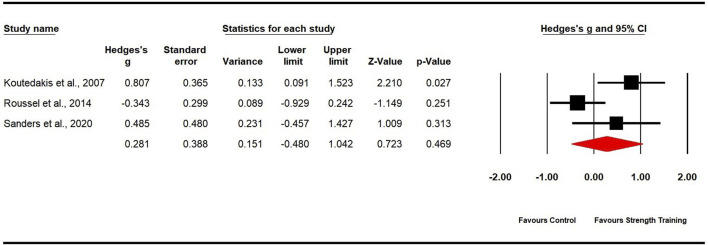
Forest plot of changes in cardiorespiratory endurance in dancers participating in strength training compared to controls. Values shown are effect sizes (Hedges’ g) with 95% confidence intervals (CI). The size of the plotted squares reflects the statistical weight of the study.

Three studies evaluated cardiorespiratory endurance, comprising three experimental groups and three control groups (pooled n = 92). The findings revealed non-significant, small effects in favor of strength training for dancers (ES = 0.28; 95% CI −0.48 to 1.04; p = 0.469; Figure 8). Moderate heterogeneity was observed (I^2^ = 68.85%), and the Egger’s test yielded a p-value of 0.538. To explore the sources of heterogeneity, we removed one outlier (Roussel et al., 2014), which adjusted the ES to 0.69 (95% CI: 0.12–1.26; p = 0.018) and reduced heterogeneity to 0%.

#### Flexibility

**FIGURE 9:**
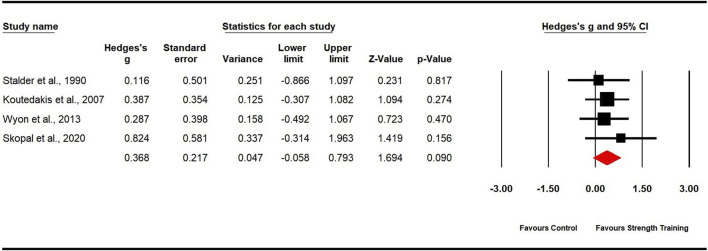
Forest plot of changes in flexibility in dancers participating in strength training compared to controls. Values shown are effect sizes (Hedges’ g) with 95% confidence intervals (CI). The size of the plotted squares reflects the statistical weight of the study.

Four studies evaluated flexibility, including four experimental groups and four control groups (pooled n = 81). The analysis indicated a non-significant, small effect in favor of strength training for dancers (ES = 0.37; 95% CI: −0.06 to 0.79; p = 0.090; Figure 9). Low heterogeneity was observed (I^2^ = 0.00%), and the Egger’s test produced a p-value of 0.615.

## Discussion

This meta-analysis evaluated peer-reviewed studies that compared the effects of strength training to control conditions on various physical fitness outcomes in dancers. The results indicated significant benefits in muscle strength and power; however, no evidence was found for positive effects on body composition, cardiorespiratory endurance, or flexibility. A detailed discussion of these findings is provided below.

### The effect of strength training on body composition in dancers

Dancers need to maintain optimal body composition to perform repetitive, high-quality, and physically demanding movements effectively (Wilmerding et al., 2005). However, the current study found no significant reductions in body mass or body fat percentage resulting from strength training. Most studies reported no changes in body mass or body fat percentage after resistance, plyometric, weight, or combined training (Koutedakis and Sharp, 2004; Brown et al., 2007; Roussel et al., 2014; Sozbir et al., 2014; De Leonardis and Greco, 2020; Sanders et al., 2020; Ávila-Carvalho et al., 2022). These findings align with a prior systematic review by Zouita et al. (2023), which concluded that resistance training does not effectively improve body composition in female athletes. In contrast, some studies suggest that strength training can be as effective as aerobic exercise in reducing body mass and body fat percentage (Carvalho et al., 2014; Wewege et al., 2022). Strength training promotes energy expenditure through muscle growth, which can aid in fat loss (Westcott, 2012).

The literature suggests that various factors can influence body composition, such as diet, nutrition, sleep, and stress (Moon, 2013; Poggiogalle et al., 2016; Stefanaki et al., 2018). Notably, experts have indicated the significant role of dietary habits and nutrition in the body composition of dancers (Chaikali et al., 2023). Dance is a physically demanding profession that requires both good athletic performance and a lean physique. Consequently, many dancers restrict their caloric intake to maintain a low body weight (Wilmerding et al., 2005). However, some researchers pointed out that the pressure to sustain low body weight and body fat, particularly in ballet, can lead to unhealthy eating patterns and health issues if not properly managed (Sousa et al., 2013). Physical activity is widely recognized as a safe means to reduce and control body fat (Zaccagni et al., 2014). Indeed, the lack of change observed in this meta-analysis may be attributed to the duration, frequency, or intensity of the training programs, as some studies suggest that interventions lasting more than 14 weeks are typically required to impact body fat and lean muscle mass (Suarez-Arrones et al., 2019; Méndez-Hernández et al., 2022). Additionally, participants in the studies may have already possessed low body fat levels, complicating the detection of any significant changes (Wilmerding et al., 2003). Accordingly, future research should aim to determine the appropriate training dose to more accurately assess its effects on muscle mass and fat reduction. To achieve a more comprehensive understanding of these changes in dancers, studies should also account for dietary factors, initial body composition, and hormonal responses (Hincapié and Cassidy, 2010; Shaw et al., 2021).

### The effect of strength training on muscle strength

Muscle strength is considered essential for improving dance performance and minimizing the risk of injuries in dancers (Koutedakis et al., 2005; Moita et al., 2017). Our findings indicate that strength training significantly improved muscle strength in dancers, closely linked to the physiological effects of the training. By increasing external resistance, strength training subjects muscle fibers to both mechanical tension and metabolic stress (Schoenfeld, 2013). This stimulation results in an increase in muscle fiber cross-sectional area (i.e., hypertrophy) and enhances strength through improved neural adaptations, specifically the efficiency and synchronization of motor neuron recruitment (Carroll et al., 2011). Moreover, strength training boosts muscles’ ability to withstand external forces, strengthening tendons and ligaments, which in turn enhances joint stability and overall force transmission efficiency (Chaudhry et al., 2017). For dancers, especially during complex movements like jumps and spins, greater muscle strength can significantly enhance body control and the precision of technical execution (Özkal et al., 2024).

In line with the findings of our meta-analysis, strength training has been well-established as an effective method for improving strength performance across a range of sports. For example, research shows that strength training significantly enhances leg strength and explosiveness in adolescent soccer players (McKinlay et al., 2018). Similarly, in basketball, strength training improves lower limb strength, contributing to enhanced jump performance and better rebounding capabilities (Yáñez-García et al., 2022). Swimmers also experience benefits from strength training, which enhances muscle strength and positively influences short-to medium-distance performance by improving force transmission and stroke mechanics (González et al., 2023). However, the analysis revealed moderate heterogeneity for muscle strength (I^2^ = 68.77%), which may be attributed to a study that included a diverse group of participants, consisting of ballet, modern, and jazz dancers (Vetter and Dorgo, 2009). This study reported an exceptionally large ES (4.15). The inclusion of participants from multiple dance styles may have influenced the training effects on muscle strength. Based on our findings, strength training appears to be effective in improving muscle strength in dancers.

### The effect of strength training on muscle power

In dance, effective utilization of muscle power is vital for executing fast and repetitive movements, such as sequences of jumps (Malkogeorgos et al., 2013). For instance, it has been reported that ballet and modern dancers land on a single limb 50% and 89% of the time, respectively (Pappas et al., 2012). This meta-analysis revealed that strength training had a significant impact on muscle power in dancers when compared to regular dancing groups. The observed increase in muscle power from the strength training intervention was expected, given the historical evidence that various strength training programs lead to power gains. For example, Mascarin et al. (2017) reported significant improvements in muscle power and transfer effects on ball speed following a 6-week strength training program for young female handball players. Similarly, Rodríguez-Rosell et al. (2017) found notable increases in muscle power after a 6-week high-speed strength training regimen for young soccer players across different age groups. A recent study by Wang et al. (2024) observed significant improvements in muscle power following an 8-week flywheel strength training program in elite female volleyball players. Furthermore, our findings align with previous meta-analyses, suggesting that incorporating strength training can enhance power adaptations (Behm et al., 2015; Slimani et al., 2018; Uysal et al., 2023)

The observed improvements in muscle power performance among dancers can be partially attributed to physiological adaptations resulting from strength training. These adaptations are largely driven by neural pruning, which enhances motor efficiency and automates movement patterns (Ahtiainen, 2019). Research indicates that the increases in muscle power stem from various muscular adaptations, including changes in muscle size and architecture, as well as variations in the mechanical stiffness of muscle-tendon structures (Bojsen-Møller et al., 2005; Duchateau et al., 2021). In this meta-analysis, jump ability serves as the primary measure of muscle power, as it is widely recognized as a strong predictor of dance performance (Angioi et al., 2009b). Additionally, several studies have incorporated plyometric exercises to further enhance dancers’ performance (Brown et al., 2007; Sozbir et al., 2014; De Leonardis and Greco, 2020; Skopal et al., 2020). These exercises improve muscular function by utilizing a quick eccentric loading phase followed by rapid concentric contraction, thereby training the stretch reflex and harnessing stored elastic energy to enhance vertical jump performance (Markovic and Mikulic, 2010). Overall, this meta-analysis confirms that strength training positively impacts muscle power in dancers.

### The effect of strength training on cardiorespiratory endurance

Good cardiorespiratory fitness is associated with quicker recovery from high-intensity intermittent exercise and a lower risk of musculoskeletal injuries in sports (Tiemens et al., 2023). However, this study found no significant increase in endurance among dancers following strength training. Notably, limited literature addresses the impact of strength training on dancers’ cardiorespiratory endurance. One study identified improvements in cardiorespiratory endurance after a 12-week regimen that combined aerobic and strength training (Koutedakis et al., 2007). In contrast, two other studies reported no significant changes in endurance following 8–16 weeks of strength training (Roussel et al., 2014; Sanders et al., 2020). Similarly, previous research on the effects of strength training on cardiorespiratory endurance has yielded inconsistent outcomes. For example, significant improvements were noted in well-trained runners undergoing strength training (Beattie et al., 2014). Additionally, a meta-analysis by Ramos-Campo et al. (2021) found that resistance circuit-based training effectively enhances cardiorespiratory endurance. Conversely, some strength training programs have demonstrated no effect on cardiorespiratory endurance in various athletes (Ignjatovic et al., 2011; Thiele et al., 2020).

The observed lack of performance gains in cardiorespiratory endurance in this meta-analysis may be attributed to the inherent nature of strength training, which primarily focuses on enhancing muscle strength and anaerobic capacity (Knuttgen, 2007). Typically, improvements in cardiorespiratory endurance necessitate moderate to high-intensity aerobic exercises that foster cardiovascular adaptations through sustained workloads (Hulke et al., 2012; Ahmed et al., 2022). Moreover, high-intensity interval training has been shown to significantly enhance cardiorespiratory endurance by alternating short periods of intense exercise with intervals of lower intensity, effectively promoting cardiovascular adaptations (Sultana et al., 2019). Additionally, the volume, frequency, and duration of the training in the included studies may not have been sufficient to elicit significant cardiovascular changes. While strength training may not directly enhance cardiorespiratory endurance, it could indirectly improve dancers’ movement efficiency and performance by increasing muscle strength and power, potentially reducing fatigue during performances (Izquierdo et al., 2006). Of note, the moderate heterogeneity (I^2^ = 68.85%) observed in this analysis may be explained by one study that combined aerobic endurance, strength, proprioception, and motor control training (Roussel et al., 2014). Such a multimodal intervention introduces variability in training protocols and participant responses, which could contribute to differences in outcomes compared to studies focusing on isolated training modalities. Collectively, further research is needed to determine the role of strength training in developing endurance performance effectively.

### The effect of strength training on flexibility

Dancers are often perceived by the public as having fit, slim physiques and a wide range of joint mobility, traits considered advantageous in various dance performances (Steinberg et al., 2018). However, the results of this meta-analysis indicate that, despite engaging in strength training, dancers did not demonstrate significant improvements in flexibility. Supporting this observation, research by Morton et al. (2011) indicated that strength-focused exercises were generally less effective than dedicated flexibility training, such as static stretching, for improving flexibility. Conflicting results have been reported in studies involving young men (Leite et al., 2017), older women (Carneiro et al., 2015), and athletes (Saraiva et al., 2014), suggesting that resistance training can sometimes lead to increases in flexibility. The differing goals and mechanisms of strength training may explain the results we observed. While the primary focus of strength training is often on increasing muscle fiber size and enhancing strength output (Suchomel et al., 2018), it typically does not prioritize joint flexibility and range of motion, which are critical for dancers. Although full-range motion exercises may provide some flexibility benefits, the strength training programs utilized in the included studies may not have delivered sufficient stimulus to enhance the dancers’ joint flexibility. Additionally, dancers typically begin with a high baseline level of flexibility (Gupta et al., 2004), which may further restrict the potential for noticeable improvement in this area.

Some researchers argue that stretching is the most frequently recommended approach for improving flexibility (Medeiros et al., 2016; Ikeda and Ryushi, 2021). Numerous studies have highlighted the positive effects of Pilates training on dancers’ flexibility (Amorim et al., 2011; Ahearn et al., 2018), underscoring the importance of flexibility-specific training methods for this group. However, our meta-analysis included a study that compared the effects of resistance training and static stretching on flexibility, finding no significant differences in outcomes between the two conditions (Morton et al., 2011). Furthermore, a recent investigation revealed that resistance training through a full range of motion can be as effective as static stretching in enhancing flexibility (Rosenfeldt et al., 2024). Overall, our findings suggested that strength training might not substantially enhance flexibility in dancers; however, further research is required to reach a definitive conclusion.

### Practical applications

The findings indicate that strength training modalities, including resistance, plyometric, weight training, and combined programs, have demonstrated positive effects on improving muscle strength and power in dancers. However, due to limited data, more specific evidence-based recommendations cannot yet be established. This meta-analysis provides preliminary guidelines, suggesting a minimum training duration of 6 weeks with 2–3 sessions per week, lasting 20–75 min per session. Exercise volume typically ranged from 2 to 10 sets per exercise, with 5–20 repetitions per set. Despite these benefits, the meta-analysis found no significant effects of strength training on body composition, cardiorespiratory endurance, or flexibility in dancers. Further research is required to confirm these findings and identify potential influencing factors. Practitioners and coaches should develop strength training programs tailored to individual competencies, aligned with dance-specific movement patterns, and scheduled well in advance of performances.

### Limitations

There are several limitations that warrant attention. Due to the limited number of studies, it was not possible to explore potential moderators such as training frequency, duration, session length, participants’ age, or dance genre. Importantly, the effects of different types of strength training may vary due to the specificity of training stimuli. However, this analysis did not compare various types of strength training, which could be an interesting and valuable direction for future research. Moreover, most studies focused on short-term interventions lasting 6–16 weeks, limiting our understanding of the long-term effects and sustainability of strength training benefits on dancers’ physical fitness. Furthermore, the studies primarily involved a limited range of dancer types, which may restrict the generalizability of the findings to other dance forms not covered in the analysis (e.g., samba, waltz). Additionally, only two studies examined the effects of neuromuscular training on dancers’ balance, and these were excluded from the meta-analysis. The positive findings reported in these studies require confirmation in future research, as balance is a vital fitness component for dancers.

## Conclusion

The findings indicate that strength modalities, such as resistance training, plyometric training, weight training, and combined programs, positively impact muscle strength and power in dancers. However, current strength training programs cannot be recommended for improving body composition, cardiorespiratory endurance, or flexibility. Future research should investigate the optimal training parameters (e.g., frequency, session length, intensity, and exercise specificity) required to enhance not only strength and power but also other aspects of physical fitness in dancers.

## Data Availability

The original contributions presented in the study are included in the article/Supplementary Material, further inquiries can be directed to the corresponding author.
