# Supplementary material for: A meta-analysis of the effects of strength training on physical fitness in dancers
Source: Front Physiol. 2025 Feb 27;16:1511833. doi: 10.3389/fphys.2025.1511833 (PMC11903753; doi:10.3389/fphys.2025.1511833)
Supplement: Supplementary file 1 [file Table1.docx]

**Detailed search strategy**

**Search on December 10, 2024**

| **Databases** | **search strategy** | **Results** |
| --- | --- | --- |
| PubMed | (danc*[Title/Abstract] OR ballet[Title/Abstract] OR salsa[Title/Abstract] OR ballroom[Title/Abstract] OR "hip hop"[Title/Abstract] OR jazz[Title/Abstract] OR tap[Title/Abstract] OR cha-cha,[Title/Abstract] OR rumba[Title/Abstract] OR samba[Title/Abstract] OR flamenco[Title/Abstract] OR tango[Title/Abstract] OR waltz[Title/Abstract] OR folk[Title/Abstract]) AND ("strength training"[Title/Abstract] OR "resistance training"[Title/Abstract] OR "weight training"[Title/Abstract] OR "power training"[Title/Abstract] OR "plyometric training"[Title/Abstract] OR "complex training"[Title/Abstract] OR "compound training"[Title/Abstract] OR "neuromuscular training"[Title/Abstract]) | 117 |
| Web of Science Core Collection | (AB=(danc* OR ballet OR salsa OR ballroom OR “hip hop” OR jazz OR tap OR cha-cha, OR rumba OR samba OR flamenco OR tango OR waltz OR folk)) AND AB=(“strength training” OR “resistance training” OR “weight training” OR “power training” OR “plyometric training” OR “complex training” OR “compound training” OR “neuromuscular training”) | 152 |
| SPORTDicus | AB (danc* OR ballet OR salsa OR ballroom OR “hip hop” OR jazz OR tap OR cha-cha, OR rumba OR samba OR flamenco OR tango OR waltz OR folk) AND AB (“strength training” OR “resistance training” OR “weight training” OR “power training” OR “plyometric training” OR “complex training” OR “compound training” OR “neuromuscular training”) | 158 |
| Scopus | (TITLE-ABS-KEY(danc* OR ballet OR salsa OR ballroom OR “hip hop” OR jazz OR tap OR cha-cha, OR rumba OR samba OR flamenco OR tango OR waltz OR folk) AND TITLE-ABS-KEY(“strength training” OR “resistance training” OR “weight training” OR “power training” OR “plyometric training” OR “complex training” OR “compound training” OR “neuromuscular training”)) | 400 |
| CINAHL | AB (danc* OR ballet OR salsa OR ballroom OR “hip hop” OR jazz OR tap OR cha-cha, OR rumba OR samba OR flamenco OR tango OR waltz OR folk) AND AB (“strength training” OR “resistance training” OR “weight training” OR “power training” OR “plyometric training” OR “complex training” OR “compound training” OR “neuromuscular training”) | 55 |
| Embase | (danc* OR ballet OR salsa OR ballroom OR “hip hop” OR jazz OR tap OR cha-cha, OR rumba OR samba OR flamenco OR tango OR waltz OR folk) AND (“strength training” OR “resistance training” OR “weight training” OR “power training” OR “plyometric training” OR “complex training” OR “compound training” OR “neuromuscular training”) | 298 |
| Cochrane Library | (danc* OR ballet OR salsa OR ballroom OR “hip hop” OR jazz OR tap OR cha-cha, OR rumba OR samba OR flamenco OR tango OR waltz OR folk) AND (“strength training” OR “resistance training” OR “weight training” OR “power training” OR “plyometric training” OR “complex training” OR “compound training” OR “neuromuscular training”) | 167 |
| Total |  | 1347 |
